# KN3014, a piperidine-containing small compound, inhibits auto-secretion of IL-1β from PBMCs in a patient with Muckle–Wells syndrome

**DOI:** 10.1038/s41598-020-70513-0

**Published:** 2020-08-11

**Authors:** Naoe Kaneko, Mie Kurata, Toshihiro Yamamoto, Tomonari Shigemura, Kazunaga Agematsu, Takashi Yamazaki, Hiroyuki Takeda, Tatsuya Sawasaki, Tomohiro Koga, Atsushi Kawakami, Akihiro Yachie, Kiyoshi Migita, Koh-ichiro Yoshiura, Takeshi Urano, Junya Masumoto

**Affiliations:** 1 Department of Pathology, Ehime University Proteo-Science Center and Graduate School of Medicine, Shitsukawa 454, Toon, Ehime 791-0295 Japan; 2 Department of Pediatrics, Shinshu University Graduate School of Medicine, Asahi 3-1-1, Matsumoto, Nagano 390-8621 Japan; 3 Department of Infectious Immunology, Shinshu University Graduate School of Medicine, Asahi 3-1-1, Matsumoto, Nagano 390-8621 Japan; 4 Department of Pediatrics and Adolescent Medicine, Tokyo Medical University, Nishishinjuku 6-7-1, Shinjuku, Tokyo 160-0023 Japan; 5 Division of Proteo-Drug-Discovery Sciences, Ehime University Proteo-Science Center, Bunkyocho 3, Matsuyama, Ehime 790-8577 Japan; 6 Division of Cell-Free Sciences, Ehime University Proteo-Science Center, Bunkyocho 3, Matsuyama, Ehime 790-8577 Japan; 7 Division of Advanced Preventive Medical Sciences, Department of Immunology and Rheumatology, Nagasaki University Graduate School of Biomedical Sciences, Nagasaki, 852-8501 Japan; 8 Center for Bioinformatics and Molecular Medicine, Nagasaki University Graduate School of Biomedical Sciences, Nagasaki, 852-8501 Japan; 9 Division of Medical Safety, Kanazawa University Hospital, Kanazawa, Ishikawa 920-8641 Japan; 10 Department of Rheumatology, Fukushima Medical University School of Medicine, Fukushima, 960-1295 Japan; 11 Department of Human Genetics, Atomic Bomb Disease Institute, Nagasaki University, 1-12-4, Nagasaki, 852-8523 Japan; 12 Department of Biochemistry, Shimane University School of Medicine, Izumo, Shimane 693-8501 Japan

**Keywords:** Drug screening, Autoinflammatory syndrome

## Abstract

NLRP3, an intracellular pattern recognition receptor, recognizes numerous pathogens and/or its own damage-associated molecules, and forms complexes with the adaptor protein ASC. These complexes constitute the NLRP3 inflammasome, a platform for processing interleukin (IL)-1β and/or IL-18. Several NLRP3 mutations result in constitutive activation of the NLRP3 inflammasome, causing cryopyrin-associated periodic syndrome (CAPS). To the best of our knowledge, small compounds that specifically inhibit inflammasome activation through the pyrin domain (PYD) have not yet been developed. This study describes an attempt to develop small compounds targeting the NLRP3 inflammasome. A core chemical library of 9,600 chemicals was screened against reconstituted NLRP3 inflammasome in a cell-free system with an amplified luminescence proximity homogeneous assay and a cell-based assay by human peripheral blood mononuclear cells (PBMCs). Inflammasome activation was evaluated by ASC-speck formation in human PBMCs, accompanied by IL-1β secretion and processing, and by using IL-1β-based dual operating luciferase (IDOL) mice. The activity of these compounds was evaluated clinically using PBMCs from a patient with Muckle–Wells syndrome (MWS), a type of CAPS, with an R260W mutation in NLRP3. Screening identified KN3014, a piperidine-containing compound targeting the interaction between NLRP3 and ASC through the PYD. KN3014 reduced ASC-speck formation in human PBMCs, luminescence from IDOL mice, and auto-secretion of IL-1β by PBMCs from the patient with MWS. These findings suggest that KN3014 may be an attractive candidate for treatment of MWS, as well as other NLRP3 inflammasomopathies.

## Introduction

Nucleotide-binding domain, leucine-rich-containing family, pyrin domain-containing-3 (NLRP3/cryopyrin), which is an intracellular pattern recognition receptor (PRR), recognizes pathogen-associated molecular pattern molecules (PAMPs) and/or danger-associated molecular pattern molecules (DAMPs) and binds apoptosis associated speck-like protein containing a caspase recruitment domain (ASC) to pro-caspase-1, thereby forming a large IL-1β processing platform called the NLRP3 inflammasome^1–3^.

NLRP3 mutations cause a hereditary autoinflammatory syndrome called cryopyrin-associated periodic syndrome (CAPS), which is characterized by periodic fever, arthralgia, and rash. Patients with CAPS can present various forms of systemic inflammatory disease, including familial cold autoinflammatory syndrome (FCAS)/familial cold urticaria (FCU), Muckle–Wells syndrome (MWS), and neonatal-onset multisystem inflammatory disease (NOMID)/chronic infantile neurologic cutaneous and articular syndrome (CINCA), in the absence of PAMP and/or DAMP stimulation^4–7^. NLRP3 has been known to be involved in the pathogenesis of metabolic diseases such as Alzheimer’s disease, atherosclerosis, gout, obesity, and type 2 diabetes^8–13^. In addition, NLRP3 may be involved in diseases of the central nervous system, lungs, liver, and kidneys, as well as in aging^14–18^. Since these diseases are thought to be caused by endogenous metabolites which can activate the NLRP3 inflammasome, the inflammasome-related disorders are currently suggested to be collectively named inflammasomopathies^19,20^.

Several inhibitors of IL-1 signaling, such as anakinra, rilonacept, and canakinumab, have been approved clinically and are effective against NLRP3-associated diseases^21^; however, to the best of our knowledge, no small inflammasome-specific compounds that target through the pyrin domain (PYD) have been developed to treat these conditions. To identify small compounds targeting the NLRP3 inflammasome through PYD, 9,600 compounds in a core chemical library established by the Drug Discovery Initiative (University of Tokyo, Tokyo, Japan) were screened against reconstituted NLRP3 inflammasome in a cell-free system^22^. The present study describes a new small compound that targets the NLRP3 inflammasome through PYD in a cell-free system. This compound could be used to treat CAPS and other NLRP3 inflammasomopathies.

## Results

### Selection of candidate NLRP3 inflammasome inhibitors by screening with reconstituted NLRP3 inflammasome in a cell-free system

**Figure 1:**
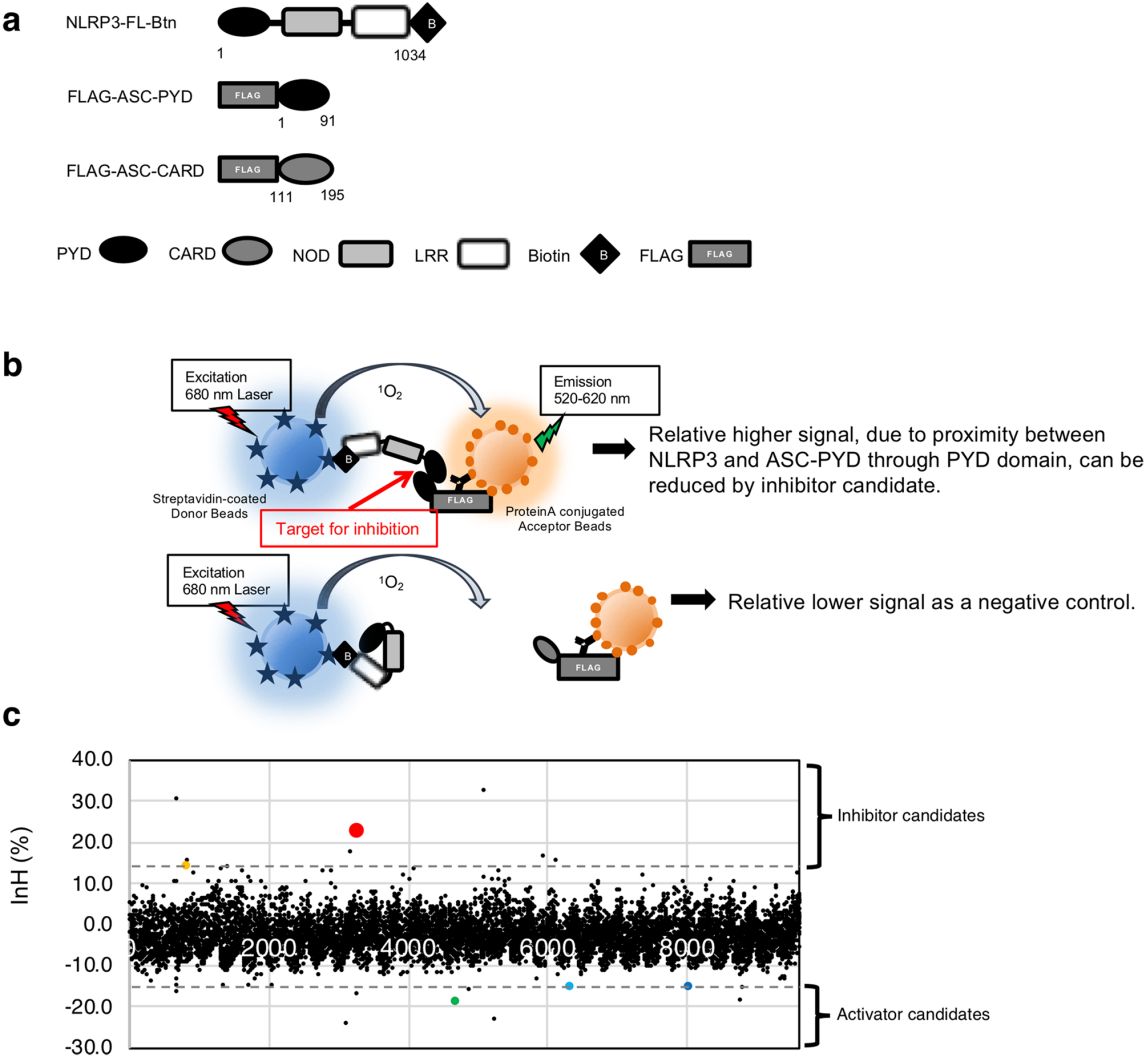
High-throughput screening of a 9,600 core chemical library using NLRP3 inflammasome in a cell-free system. (**a**) Schematic representations of C-terminal biotinylated full-length NLRP3 (NLRP3-FL-Btn), the N-terminal FLAG-tagged PYD of ASC (FLAG-ASC-PYD), and the N-terminal FLAG-tagged caspase recruit domain (CARD) of ASC (FLAG-ASC-CARD). *PYD* pyrin domain, *CARD* caspase recruitment domain, *NOD* nucleotide-binding oligomerization domain, *LRR* leucine rich repeat. (**b**) Schematic representation of reconstituted inflammasomes. The PYD of truncated ASC was able to open and bind to the PYD of NLRP3. The chemical energy of the reactive oxygen on donor beads was transferred to acceptor beads, and a signal was detected. (**c**) Primary screening of the 9,600 core chemical library using NLRP3 inflammasome in a cell-free system. The result presented was the only result obtained.

The amplified luminescence proximity homogeneous assay (ALPHA)-screen-based chemical library of 9,600 compounds was subjected to high-throughput screening using reconstituted NLRP3 inflammasome in a cell-free system^22,23^ (Fig. 1a,b and Supplementary Figs. S1, S2, and S3). The sample layout is shown schematically (Supplementary Fig. S2). Screening did not involve use of a combination of constitutively active mutant C-terminal biotinylated full-length NLRP3 (NLRP3-FL-Btn) and FLAG-ASC-FL for the constructs, or a combination of wild-type NLRP3-FL-Btn and FLAG-ASC-FL with ligand, because a truncated form of ASC, containing a PYD but lacking a caspase recruitment domain (CARD), was able to interact with NLRP3 in the absence of NLRP3 ligand^22^. By contrast, a truncated form of ASC, containing a CARD but lacking a PYD, was unable to interact with NLRP3^22^. The positive control consisted of a combination of NLRP3-FL-Btn and FLAG-ASC-PYD (57,237 counts), and the negative control consisted of a combination of NLRP3-FL-Btn and FLAG-ASC-CARD (922 counts). The quality of high-throughput screening of the ALPHA-screen-based chemical library screening was assessed by calculating the z-factor as follows: z = 1 − [3 × (standard deviation of positive controls) + 3 × (standard deviation of negative controls)]/[(average of positive controls) − (average of negative controls)].

In general, assays with a z-factor greater than 0.5 are considered accurate and suitable for high-throughput screening. The calculated z-factor was 0.87. Screening was performed at a final compound concentration of 1.0 µM. The inhibition rate (InH) (%) was calculated as 100 × {1 − (value of sample − value of negative control)/(value of positive control − value of negative control)}. The InH (%) of each compound is shown in a dot plot (Fig. 1c). Compounds with ± 15% InH were selected for further screening; these included two candidate inhibitors, KN3014 and KN8311, and three negative controls, KN1960, KN5330, and KN7644. The five selected compounds (KN3014, KN8311, KN1960, KN7644, and KN5330) are depicted by red, orange, green, blue, and dark blue dots, respectively (Fig. 1c).

### Secondary screening of selected compounds by titration against reconstituted NLRP3 inflammasome in a cell-free system

The five selected compounds were subjected to secondary screening by quadruplicate screening against NLRP3 inflammasome (Supplementary Fig. S3). The interaction between NLRP3-FL-Btn and FLAG-ASC-PYD was disrupted by both KN3014 and KN8311 in a dose-dependent manner. KN3014 and KN8311 had maximum InH (%) values of 56.42% and 39.24%, respectively, and IC50 values of 14.65 and 118.29 μM, respectively (Supplementary Table S1). By contrast, neither KN5330 nor KN7644 had an effect on the interaction between NLRP3-FL-Btn and FLAG-ASC-PYD. The maximum InH (%) of KN1960 was − 30.80%, promoting the interaction between NLRP3-FL-Btn and FLAG-ASC-PYD.

### Cell-based screening of the five compounds with peripheral blood mononuclear cells

**Figure 2:**
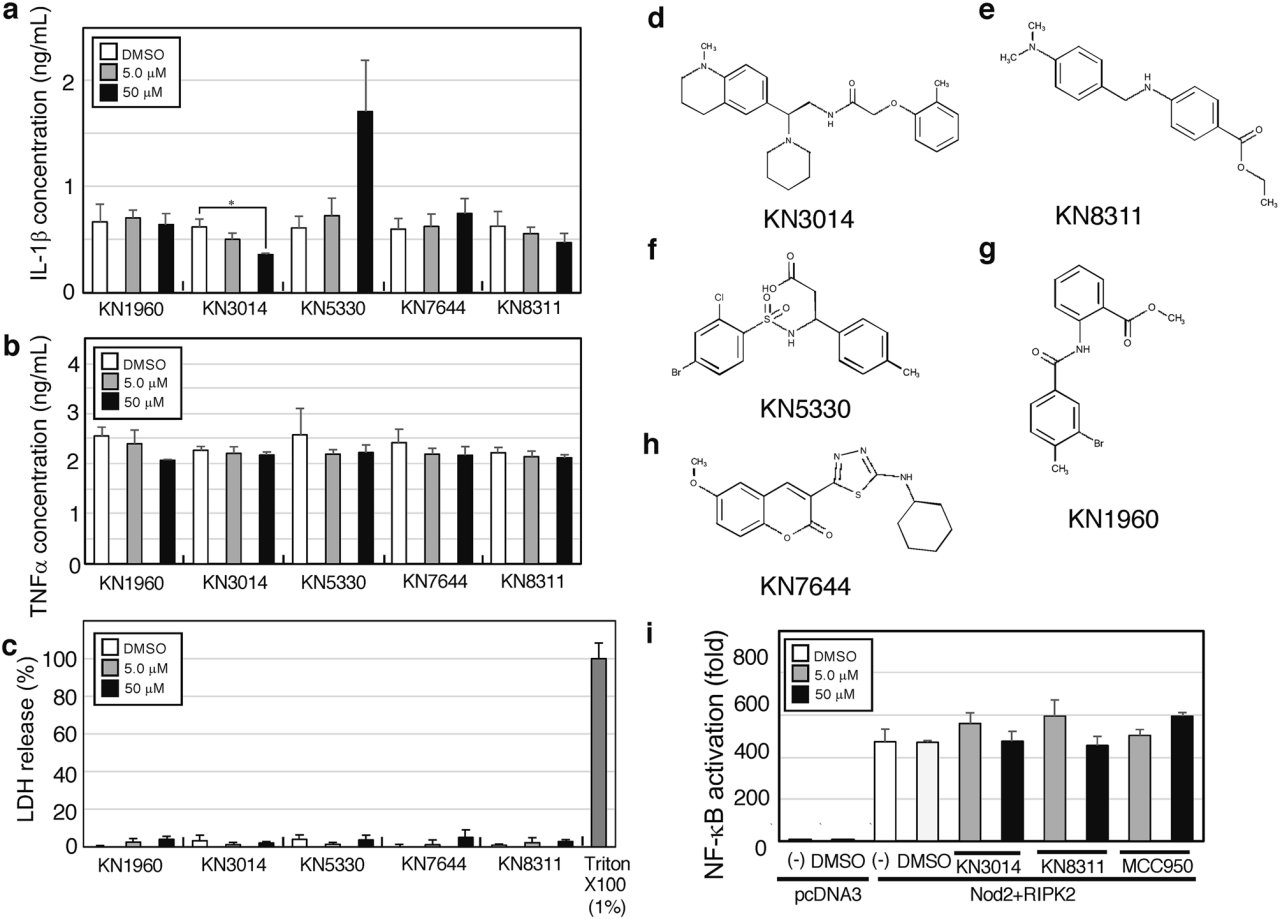
Cell-based screening using human peripheral mononuclear cells and the chemical structure of compounds targeting the NLRP3 inflammasome. A total of 1 × 10^5^ human PBMCs were incubated with 0.1 ng/mL LPS for 8 h. (**a**) IL-1β concentrations in the culture supernatant measured by ELISA. (**b**) TNF-α concentrations in the culture supernatant were measured by ELISA. (**c**) LDH concentrations in the culture supernatant measured by CytoTox (Promega). (**d–h**) Chemical structures of (**d**) KN3014, (**e**) KN8311, (**f**) KN5330, (**g**) KN1960, and (**h**) KN7644. (**i**) NF-κB luciferase reporter gene assay. A total of 1 × 10^5^ human embryonic kidney 293T cells were transfected with 33 ng of pcDNA3-Nod2-FLAG and 33 ng of pcDNA3-RIPK2-myc or 66 ng of pcDNA3 (Vector), or were left untreated (−). Eight hours later, the medium was replaced with DMEM containing the indicated concentrations of KN3014, KN8311, and MCC950, and the cells were incubated for 16 h. (**a**–**c**) The results were obtained from cell-based screening. The column in each figure is presented as the mean ± standard deviation of triplicate cultures and are representative of two independent experiments. *p-value < 0.05 (Mann–Whitney *U*-test). (**i**) Results are presented as the mean ± standard deviation of triplicate measurements and are representative of three independent experiments.

The ability of these five compounds to affect cytokine secretion by lipopolysaccharide (LPS)-stimulated human peripheral blood mononuclear cells (PBMCs) was assessed by cell-based screening with each compound tested in triplicate wells. Briefly, 1 × 10^5^ human PBMCs were stimulated with 0.1 ng/mL LPS in the presence of 5.0 or 50 μM of one of the selected compounds or DMSO for 8 h, and the concentrations of IL-1β (Fig. 2a) and TNF-α (Fig. 2b) in the culture supernatants were measured in an enzyme-linked immunosorbent assay (ELISA). KN3014 was the only compound that markedly inhibited IL-1β secretion from PBMCs but had no effect on TNF-α secretion without serious cytotoxicity (Fig. 2c). KN8311 slightly reduced IL-1β secretion but had no effect on TNF-α secretion without serious cytotoxicity.

### Structures of KN3014, KN8311, KN5330, KN1960, and KN7644

Based on these screening results, clarified chemical structures of KN3014, KN8311, KN5330, KN1960, and KN7644 from the Drug Discovery Initiative (University of Tokyo, Tokyo, Japan) were determined. KN3014 was identified as *N*-(2-(1-methyl-1,2,3,4-tetrahydroquinolin-6-yl)-2-(piperidin-1-yl)ethyl)-2-(o-tolyloxy)acetamide, with a molecular formula of C_26_H_35_N_3_O_2_ and a molecular weight of 422 daltons (Fig. 2d). KN8311 was identified as ethyl-4-((4-(dimethylamino)benzyl)amino) benzoate, with a molecular formula of C_18_H_22_N_2_O_2_ and a molecular weight of 298 daltons (Fig. 2e). KN5330 was identified as 3-(4-bromo-2-chlorophenylsulfonamido)-3-p-tolylpropanoic acid, with a molecular formula of C_16_H_15_BrClNO_4_S and a molecular weight of 433 daltons (Fig. 2f). KN1960 was identified as methyl 2-((3-bromo-4-methylbenzoyl)amino)benzoate, with a molecular formula of C_16_H_14_BrNO_3_ and a molecular weight of 348 daltons (Fig. 2g). KN7644 was identified as 3-(5-(cyclohexylamino)-1,3,4-thiadiazol-2-yl)-6-methoxy-2H-chromen-2-one, with a molecular formula of C_18_H_19_N_3_O_3_S and a molecular weight of 357 daltons (Fig. 2h).

### Neither KN3014 nor KN8311 affected Nod2 + RIPK2-induced NF-κB activation

To determine whether KN3014 and KN8311 affect the NOD2-RIPK2-induced NF-κB activation pathway, we employed the NF-κB reporter gene assay involving Nod2 with RIPK2. Co-transfection of human embryonic kidney (HEK) 293 T cells with 33 ng of pcDNA3-Nod2-FLAG and 33 ng of pcDNA3-RIPK2-myc induced approximately 400-fold higher NF-κB activation than transfection of these cells 66 ng of the vector pcDNA3. NF-κB activation was not affected by either KN3014 or KN8311 at concentrations of 5.0 or 50 μM, or by the NLRP3 inhibitor control MCC950 at the same concentrations (Fig. 2i).

### KN3014 reduced ASC-speck formation accompanied by IL-1β processing and secretion without affecting LPS-induced pro-IL-1β production

**Figure 3:**
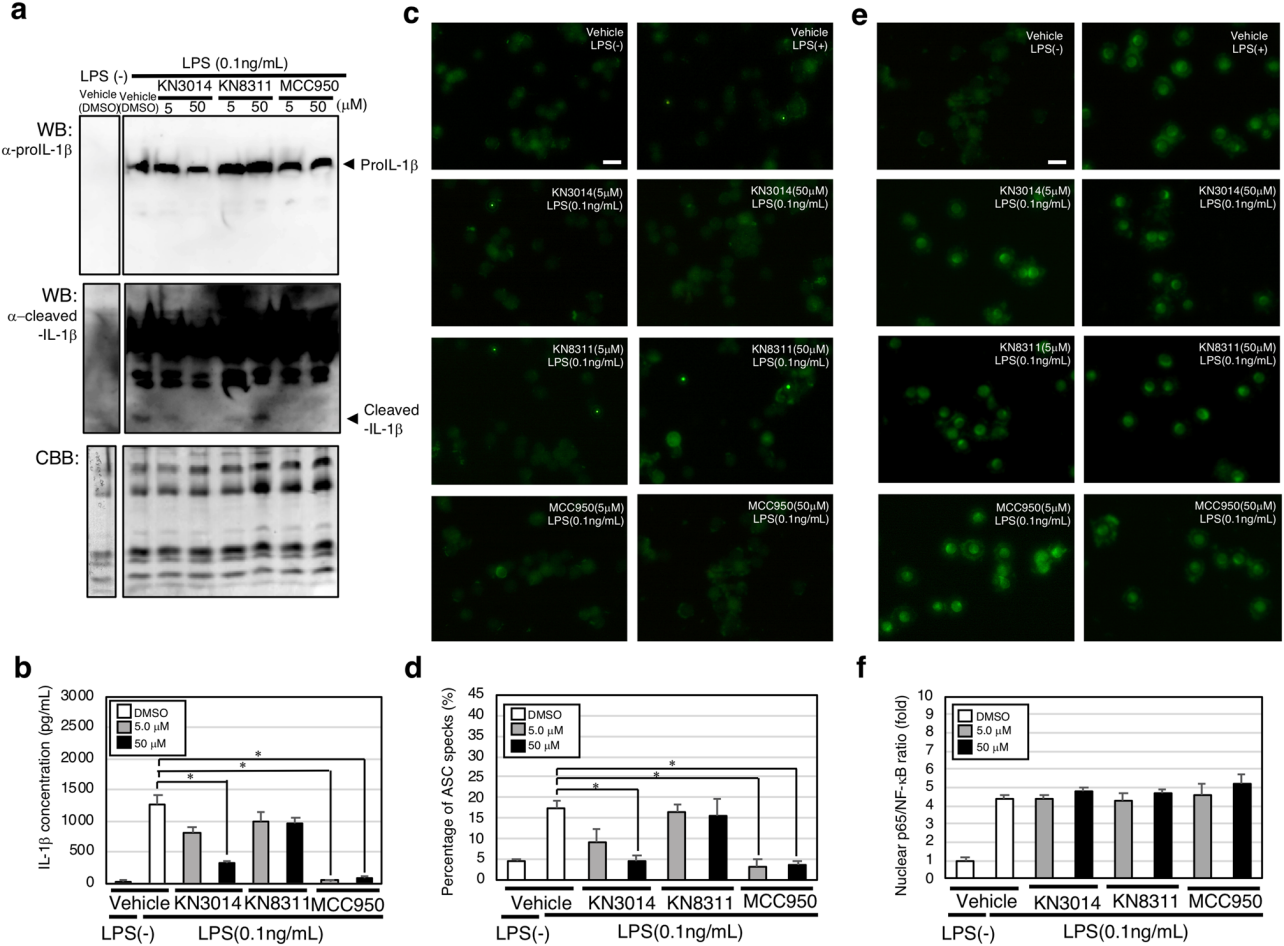
KN3014 reduced ASC-speck formation accompanied by IL-1β processing and secretion with no effect on pro-IL-1β production or NF-κB activation. (**a**) Western blotting analysis of LPS (0.1 ng/mL) induced pro-IL-1β production (upper panel) and IL-1β cleavage (middle panel; the same blotting membrane shown in the upper panel was re-hybridized with a mAb specific for cleaved-IL-1β (long exposure)). The same gel was stained with Coomassie brilliant blue (lower panel). (**b**) IL-1β concentrations in the supernatant of each well measured by ELISA. (**c**) Immunofluorescence microscopy evaluation of ASC-speck formation. (**d**) Percentage of ASC specks (%). (**e**) Immunofluorescence microscopy evaluation of NF-κB p65 nuclear translocation. (**f**) Relative nuclear translocation of NF-κB p65. Results are given as means ± standard deviation of three hyper views. *p-value < 0.05 (Mann–Whitney *U*-test). These results are representative of two independent experiments. Scale bars = 10 μm.

We tested whether KN3014 or KN8311 could inhibit inflammasome activation in LPS-stimulated human PBMCs. First, we evaluated whether KN3014 or KN8311 could inhibit IL-1β processing and secretion, finding that the induction of pro-IL-1β in PBMCs treated with 0.1 ng/mL LPS was unchanged by KN3014, KN8311, or MCC950 (Fig. 3a, upper panel), whereas processing of IL-1β was inhibited by KN3014 in a dose-dependent manner (Fig. 3a, middle panel), without affecting protein expression (Fig. 3a, lower panel). The concentrations of IL-1β in the supernatants of PBMCs cultured with 0.1 ng/mL LPS were dose-dependently reduced by 5.0 and 50 μM KN3014, but not by KN8311. In comparison, 5.0 and 50 μM MCC950 completely inhibited the secretion of IL-1β into culture supernatants (Fig. 3b).

Because ASC-speck formation is regarded as a simple upstream readout of inflammasome activation^24^, we assessed the effects of KN3014, KN8311, and MCC950 on ASC-speck formation. Consistent with their effects on IL-1β processing, KN3014 and MCC950 dose-dependently reduced ASC-speck formation by 0.1 ng/mL LPS-treated PBMCs, whereas KN8311 had no effect (Fig. 3c,d). None of these compounds had an effect on NF-κB p65 nuclear translocation, as observed by immunofluorescence microscopy (Fig. 3e,f).

### KN3014 reduced inflammasome activation in IL-1β-based dual operating luciferase mice

**Figure 4:**
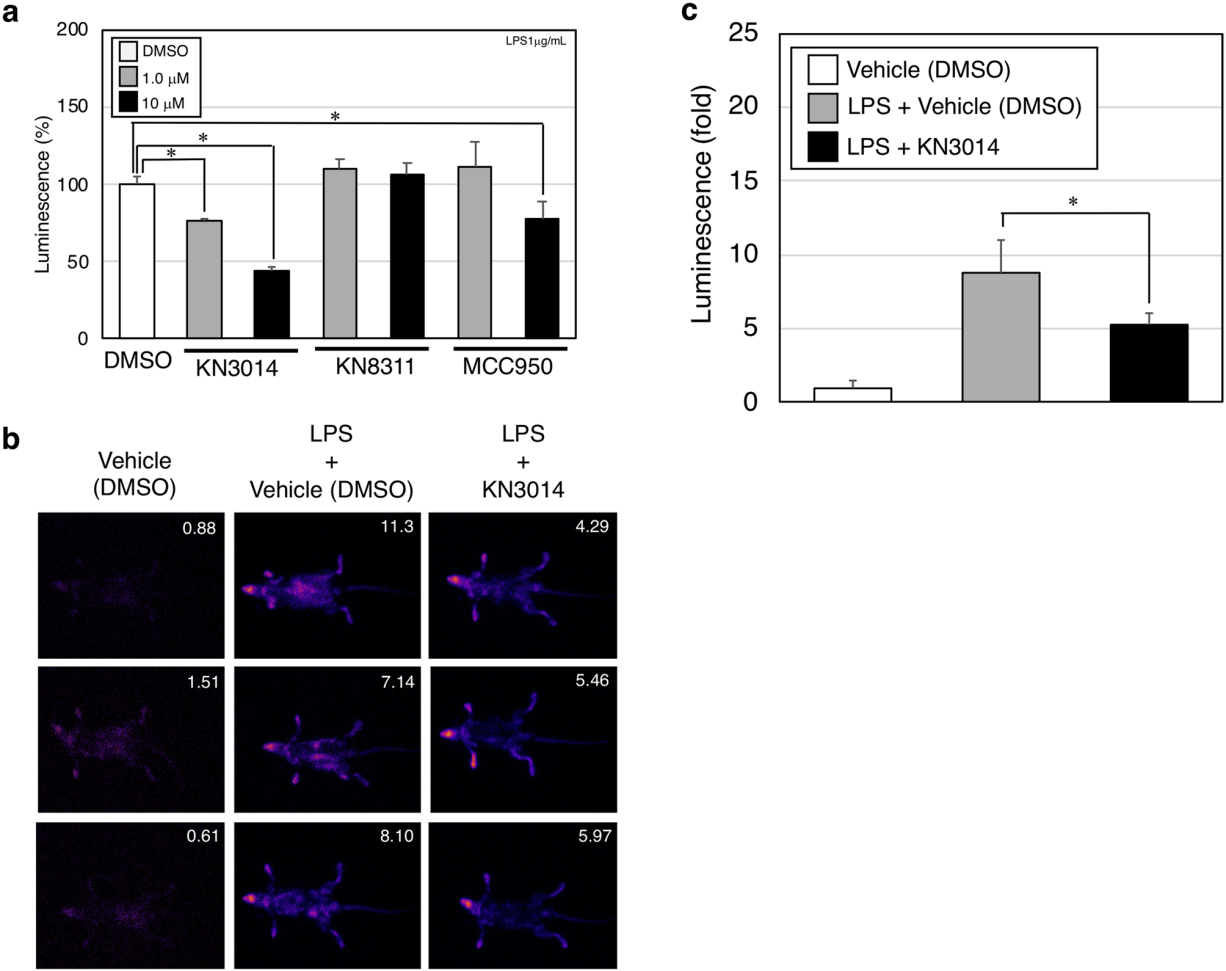
Inhibition of luciferase luminescence in IL-1β-based dual operating luciferase mice. (**a**) A total of 1 × 10^6^ splenocytes obtained from the resected spleen of an IDOL mouse were incubated with 1 μg/mL LPS with or without the indicated concentrations of KN3014, KN8311, MCC950, or DMSO. Luciferin luminescence signals from splenocytes were measured. Results are given as means ± standard deviation of triplicate culture and are representative of three independent experiments. *p-value < 0.05 (Mann–Whitney *U*-test). These results are representative of three independent experiments. (**b**) In vivo imaging analysis at the whole-body level of luminescent signals from LPS-treated IDOL mice. The values of luminescence signals of individual mice are shown in the upper right corner of each panel. (**c**) Mean ± standard deviation of luminescence signal for all individual mice in each group in Fig. 4b. *p-value < 0.05 (Mann–Whitney *U*-test). These results are representative of two independent experiments.

We further tested whether KN3014 and KN8311 could inhibit inflammasome activation in a mouse model, using IL-1β-based dual operating luciferase (IDOL) mice to image IL-1β-related inflammation. Mouse splenocytes were incubated with 1.0 μg/mL LPS, in the presence of 1.0 or 10 μM KN3014 or KN8311. Both concentrations of KN3014 dose-dependently reduced the fluorescence signal from splenocytes, as did 10 μM MCC950, whereas KN8311 had no effect (Fig. 4a). Intraperitoneal injection of 0.1 μg/g body weight of LPS and 1.0 μmol/g body weight KN3014 reduced system fluorescence signal compared with intraperitoneal injection of LPS and vehicle (DMSO) (Fig. 4b,c).

### KN3014 reduced spontaneous IL-1β secretion from PBMCs of a patient with Muckle–Wells syndrome, a cryopyrin-associated periodic fever syndrome

**Figure 5:**
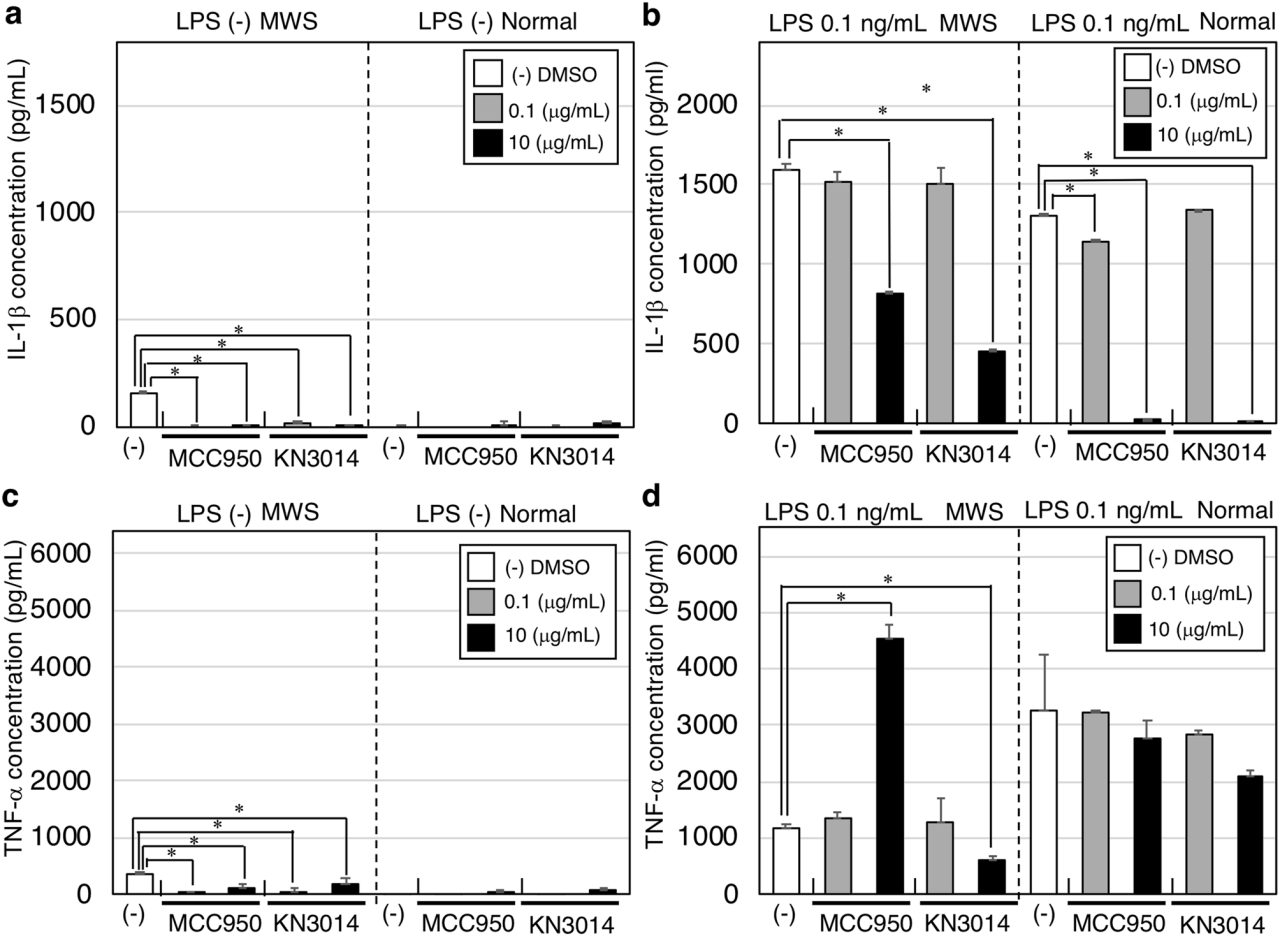
Effects of KN3014 or MCC950 on cytokine secretion from peripheral blood mononuclear cells from a patient with Muckle–Wells syndrome. A total of 1 × 10^6^ PBMCs obtained from the patient with MWS or a healthy volunteer were left untreated (−), or were treated with the indicated concentrations of KN3014 or MCC950 in the absence (**a**, **c**) or presence (**b**, **d**) of LPS for 8 h. IL-1β (**a**, **b**) and TNF-α (**c**, **d**) concentrations were measured by ELISA. *p-value < 0.05 (Mann–Whitney *U*-test). The result presented was the only result obtained.

To investigate the possible clinical activity of KN3014, we assessed whether this compound could ameliorate symptoms in a patient with CAPS. This patient was a 21-year-old Japanese woman diagnosed with MWS at age 7 years, with an arginine-to-tryptophan mutation at position 260 (R260W) of NLRP3^25^. She was being treated with injections of 150 mg canakinumab every 2 months. A blood sample was collected immediately before canakinumab administration. PBMCs isolated from this patient and from a healthy volunteer were incubated with or without 0.1 ng/mL LPS. The PBMCs from the MWS patient spontaneously secreted IL-1β, with secretion enhanced by incubation with 0.1 ng/mL LPS, whereas the PBMCs from the healthy volunteer required LPS treatment for IL-1β secretion (Fig. 5a,b). Incubation of the patient’s PBMCs with either KN3014 or MCC950 at concentrations of 0.1 μg/mL and 10 μg/mL almost completely inhibited the spontaneous secretion of IL-1β secretion (Fig. 5a) and completely inhibited IL-1β secretion by PBMCs from the healthy volunteer (Fig. 5b). The PBMCs from the patient with MWS also spontaneously secreted TNF-α in the absence of LPS, whereas the PBMCs from the healthy volunteer did not (Fig. 5c). LPS stimulated TNF-α secretion from PBMCs of the patient with MWS and the healthy volunteer, with the amount of TNF-α secreted being lower in culture supernatants of PBMCs from the patient with MWS than the healthy volunteer (Fig. 5d). KN3014 dose-dependently reduced TNF-α secretions from PBMCs of the patient with MWS and the healthy volunteer, whereas MCC950 dose-dependently enhanced TNF-α secretion from PBMCs of the patient with MWS (Fig. 5d).

### KN3014 may interfere with ASC function

Finally, we assessed whether KN3014 could inhibit ASC function. FLAG-ASC-PYD and NLRP3-FL-Btn, NLRP3-PYD-Btn, full-length AIM2-Btn (AIM2-FL-Btn), or the PYD of AIM2-Btn (AIM2-PYD-Btn) were incubated for 24 h with anti-FLAG monoclonal antibody (mAb) M2 (5 μg/mL), protein-A-conjugated ALPHA acceptor beads (16.67 μg/mL), and streptavidin-conjugated ALPHA donor beads (16.67 μg/mL), with or without 5.0 or 50 μM KN3014 or MCC950. KN3014 interfered with the interactions of FLAG-ASC-PYD with NLRP3-FL-Btn, NLRP3-PYD-Btn, AIM2-FL-Btn, and AIM2-PYD-Btn (Supplementary Fig. S4a–d), suggesting that KN3014 may interfere with ASC function through PYD (Supplementary Fig. S5a–b).

## Discussion

Activation of the NLRP3 inflammasome has been associated with the pathology of various diseases and with physiological aging^18^. Although innate defenses against pathogens based on the NLRP3 inflammasome–IL-1β axis are thought to be important, excess activation can lead to cell and tissue damage^21^. Therapeutic agents targeting IL-1β, such as anakinra, rilonacept, and canakinumab, have been used to treat patients with autoinflammatory diseases, such as CAPS, which involve gain of function mutations of NLRP3; however, IL-1β-targeting biologic agents affect various types of signals from inflammasomes and/or non-specific proteases that result in the accumulation of IL-1β^21^. Patients with CAPS and/or inflammasomopathies require treatment with small compounds that target the inflammasome. The present study describes a piperidine-containing small compound that targets the interaction between NLRP3 and ASC through PYD. This compound also inhibited the constitutive auto-secretion of IL-1β by PBMCs from a patient with MWS.

A total of 9,600 compounds were screened to identify those that could reduce the interaction between NLRP3 and the PYD of ASC, called the NLRP3 inflammasome, in a cell-free system with ALPHA^22^ (Fig. 1a–c and Supplementary Figs. S1, S2, and S3). Because this reconstituted NLRP3 inflammasome is highly customized for drug discovery based on protein–protein interactions between the PYDs of NLRP3 and ASC, the identified candidates may be highly specific inhibitors targeting the NLRP3 inflammasome itself (Fig. 1a,b). Previous drug screening using ALPHA in the Proteo-Science Center of Ehime University identified small compounds with high InH (> 50%) that could disrupt a broad range of interactions non-specifically. Two compounds, KN3014 and KN8311, targeting the NLRP3 inflammasome were selected. As controls for secondary screening, three additional compounds, KN1960, KN5330, and KN7644, with InH values (< 0%) were selected (Fig. 1c).

Titration experiments on secondary screening revealed that both KN3014 and KN8311 inhibited cell-free reconstituted NLRP3 inflammasome in a dose-dependent manner. Because KN3014 has an IC50 of approximately 14.65 μM, it may have clinical potential. By contrast, the IC50 of KN8311 was approximately 118.29 μM even in the cell-free model. Because KN5330 and KN7644 did not show functional curves, and KN1960 was likely to act as a ligand of the NLRP3 inflammasome, these compounds were not further analyzed (Supplementary Fig. S3 and Supplementary Table S1).

Cell-based screening using normal human PBMCs found that 50 μM KN3014 strongly inhibited IL-1β secretion, consistent with the results of primary and secondary screening with the NLRP3 inflammasome in the cell-free system. By contrast, 50 μM KN3014 did not inhibit TNF-α secretion by LPS-stimulated human PBMCs without serious cytotoxicity (Fig. 2a–c).

KN3014 and KN8311 each contain an internal methyl phenyl amine, which may be essential for targeting the NLRP3 inflammasome, with KN3014 containing a piperidine structure (Fig. 2d–h). Similar to the NLRP3 inflammasome inhibitor MCC950, neither KN3014 nor KN8311 affected Nod2- and RIPK2-dependent or intrinsic NF-κB activation, suggesting that neither KN3014 nor KN8311 affected the secretion of other cytokines induced by NF-κB, such as TNF-α (Fig. 2i).

Next, we tested whether KN3014 and KN8311 could inhibit inflammasome activation. Because ASC-speck formation with IL-1β processing is an indicator of inflammasome activation ^24^, both immunoblotting and immunofluorescence microscopy were utilized to evaluate ASC-speck formation. KN3014 reduced ASC-speck formation, accompanied by IL-1β processing and secretion, without affecting LPS-induced pro-IL-1β production or activation of NF-κB (Fig. 3a–f). This finding confirmed that KN3014, as well as MCC950, could inhibit inflammasome activation.

We also tested whether KN3014 or KN8311 could ameliorate IL-1β-dependent systemic inflammation in an animal model. Consistent with the above results, both KN3014 and MCC950 dose-dependently reduced the luminescence of splenocytes obtained from IDOL mice incubated with 1 μg/mL LPS; however, KN8311 had no effect. KN3014 also reduced systemic luminescence of splenocytes from IDOL mice treated with LPS (Fig. 4a–c). The results suggest that KN3014 actually inhibits the NLRP3 inflammasome, ameliorating IL-1β-dependent systemic inflammation in this animal model.

Residual signals were observed in IDOL mice treated with maximum concentrations of KN3014 and MCC950. LPS activation of the mouse IL-1β promoter fused to the luciferase-IDOL-construct in IDOL mice can result in the detection of signal, even when the luciferase-IDOL construct is spliced to the inflammasome. However, the luminescence signal of the unspliced form may persist. The difference between human and mouse sequences of the NLRP3 inflammasome may result in a residual signal, even after treatment with KN3014 or MCC950 (Supplementary Fig. S1).

Finally, we incubated KN3014 with PBMCs obtained from a patient with MWS. Because this patient had last been treated with canakinumab 2 months earlier, the serum concentration of canakinumab had decreased to the extent that IL-1β secretion from her PBMCs had increased. In the absence of stimulation, PBMCs from this patient secreted IL-1β and TNF-α. Both KN3014 and MCC950 dose-dependently reduced the spontaneous auto-secretion of both IL-1β and TNF-α, suggesting that KN3014 may be a candidate therapeutic agent for the treatment of patients with CAPS (Fig. 5a–d).

We previously reported that PBMCs from this patient with MWS spontaneously secreted IL-1β, whereas TNF-α secretion was inhibited even when these PBMCs were stimulated with LPS^25^. Because this patient was receiving the anti-IL-1β agent with canakinumab, the secretion of TNF-α by her PBMCs may indicate a dysfunction in a negative feedback loop^25^.

PBMCs from this patient showed a greater response to LPS stimulation than PBMCs from a normal volunteer. Secretion of IL-1β and TNF-α was not completely inhibited by 10 μg/mL KM3014 or MCC950, although both appeared effective in this MWS patient with an R260W NLRP3 mutation. These findings were consistent with results showing that MCC950 was effective in a mouse model of CAPS^26^.

Interestingly, MCC950 dose-dependently altered TNF-α secretion from LPS-stimulated PBMCs from the patient with MWS but not from the normal volunteer (Fig. 5d), suggesting that TNF-α secretion may be a tradeoff between IL-1β secretion and TNF-α secretion in response to an NLRP3 inflammasome-dependent mechanism^25^. KN3014 did not markedly affect TNF-α secretion, suggesting that the mechanisms of inhibition by KN3014 and MCC950 differ.

Indeed, KN3014 targets the NLRP3 inflammasome by inhibiting the interaction between the PYD domains of NLRP3 and ASC, whereas MCC950 targets the Walker B ATP-hydrolysis motif located in the nucleotide-binding oligomerization domain^27,28^. This difference in target site may explain the difference in physiological responses to KN3014 and MCC950, with KN3014 interfering with ASC function (Supplementary Figs. S4 and S5). The results provide new insight into mechanisms regulating the NLRP3 inflammasome.

In summary, this study identified KN3014 as a new small compound targeting the interaction between NLRP3-PYD and ASC-PYD of the NLRP3 inflammasome. Identification of this compound could lead to potential new therapeutic agents for patients with CAPS, as well as offer new insights into the mechanisms regulating the NLRP3 inflammasome and its inflammasomopathies.

## Methods

### Plasmid construction

The wheat germ cell-free protein expression plasmid clones, pEU-E01-NLRP3-FL-Bls, pEU-E01-FLAG-ASC-PYD, and pEU-E01-FLAG-ASC-CARD, were constructed as described^22,29^ and used with a WEPRO1240 Expression Kit (Cell-free Science, Matsuyama, Japan) to synthesize specific proteins, which were identified by western blotting. The mammalian expression plasmid clones pcDNA3-Nod2-FLAG and pcDNA3-RIPK2-myc were constructed as described^30^.

### Amplified luminescence proximity homogeneous assay

ALPHAs were performed as described^22^ to assess the interactions of synthesized proteins. Briefly, 100 ng of each protein was added to ALPHA buffer (100 mM Tris–HCl [pH 8.0]), 0.01% v/v Tween 20, 1 mg/mL BSA, 17 μg/mL streptavidin-conjugated ALPHA donor beads (PerkinElmer, Waltham, MA, USA), 17 μg/mL protein-A-conjugated ALPHA acceptor beads, and 5 μg/mL anti-FLAG mAb M2, and incubated in an Optiplate-384 plate (PerkinElmer) at 25 °C for 24 h. The fluorescence emission signals of each well were measured using an EnVision Multimode Plate Reader (PerkinElmer)^22^.

### Chemical library screening

Details of the screening of the ALPHA screen-based chemical library are schematically described and depicted in Fig. 1. In brief, 9,600 synthesized chemicals established by the Drug Discovery Initiative (University of Tokyo, Tokyo, Japan) were dissolved in DMSO, and 250 nL of each, at a concentration of 120 μM, was added to each well of an Optiplate-384. To each well was added 9.8 µL of the NLRP3-FL mixture containing 1 µL biotinylated NLRP3-FL using a FlexDrop dispenser (PerkinElmer). In addition, 10 µL of an ASC-PYD mixture containing 1 µL FLAG-ASC-PYD was added to sample wells and positive control wells using a FlexDrop dispenser, and 10 µL of an ASC-CARD mixture containing 1 µL FLAG-ASC-CARD was added to negative control wells using a Picus NxT dispenser (Sartorius, Goettingen, Germany). Subsequently, 10 µL of a mixture of donor beads and acceptor beads was added to each well using a FlexDrop dispenser (PerkinElmer). Each reaction mixture contained 0.833% DMSO. Each 384-well plate included 16 + 16 negative control wells (containing biotinylated NLRP3-FL and FLAG-ASC-CARD) and 16 + 16 positive control wells (containing biotinylated NLRP3-FL and FLAG-ASC-PYD). After incubating the plate at 25 ℃ for 24 h, the luminescence in each well was analyzed using the ALPHA-screen detection program.

### NF-κB reporter gene assay

HEK 293T cells were maintained in DMEM (Thermo Fisher Scientific, Waltham, MA, USA), supplemented with 10% heat-inactivated fetal bovine serum (FBS), penicillin, and streptomycin. Transfection was performed using calcium phosphate methods; briefly, plasmids were diluted in 220 μL distilled water plus 30 μL of 2 M CaCl_2_ mixed with 250 μL of 2 × HEPES buffer [50 mM HEPES (pH 7.0), 280 mM NaCl, 1.5 mM Na_2_HPO_4_], and 160 μL of each mixture was added to each 1 mL 293T cell culture in three wells. A total of 1 × 10^5^ HEK293T cells were transfected with or without the following expression plasmids: 33 ng of pcDNA3-Nod2-FLAG and 33 ng of pcDNA3-RIPK2-myc together with reporter plasmids, consisting of 8.3 ng of NF-κB-dependent pBxVI-luc reporter and 8.3 ng of pGL4.74 [hRluc/TK]. NF-κB luciferase reporter activity was measured 24 h after transfection using the GloMax Explorer System with Dual-Luciferase Reporter Assay System according to the manufacturer’s instructions (Promega, Madison, WI, USA). Values were normalized to those of firefly (Renilla) luciferase activity.

### Cytotoxicity assay

Compound cytotoxicity was assessed using CytoTox96 non-radioactive cytotoxicity assays (Promega), which measure the release of lactate dehydrogenase (LDH). LDH concentration was measured based on the absorbance of the solution at 490 nm.

### Criteria supporting inflammasome activation

Because ASC-speck formation is regarded as a simple upstream readout for inflammasome activation, the ratio of ASC-speck formation was evaluated as a criterion supporting inflammasome activation^24^. Human PBMCs were incubated with 0.1 ng/mL LPS together with KN3014, KN8311, MCC950, or vehicle (DMSO) for 8 h. LPS-induced pro-IL-1β and cleavage of IL-1β were evaluated by western blotting using a rabbit anti-IL-1β (D3U3E) mAb #12703 (Cell Signaling Technology, Danvers, MA, USA) and a rabbit anti-cleaved-IL-1β (Asp116) (D3A3Z) mAb #83186 (Cell Signaling Technology), respectively. IL-1β concentrations in the supernatant of each well were measured in an ELISA. The cells in each well were fixed on glass slides and incubated with anti-ASC mouse mAb^31^, followed by Alexa Fluor 488 AffiniPure F(ab′)_2_ fragment goat anti-mouse IgG (H + L) (Jackson ImmunoResearch, West Grove, PA, USA) and monitoring by immunofluorescence microscopy to evaluate ASC-speck formation. Alternatively, the cells were incubated with a rabbit anti-NF-κB p65 (D14E12) XP mAb #8242 (Cell Signaling Technology), followed by Alexa Fluor 488 AffiniPure F(ab′)_2_ fragment goat anti-rabbit IgG (H + L) (Jackson Immuno Research), and then monitoring by immunofluorescence microscopy to evaluate nuclear translocation of NF-κB.

### IL-1β-based dual operating luciferase mice

Inflammasome activation in vivo was evaluated using IDOL mice (TansGenic Inc., Kobe, Japan)^32^. The IDOL gene contains a mouse IL-1β promoter, luciferase, mouse IL-1β partial cDNA, and the CL1-PEST degradation signal in frame^32^. In the presence of an inflammatory signal, IL-1β reporter activity is induced. A combination anesthetic was prepared, consisting of 0.75 mL medetomidine, 2.0 mL midazolam, 19.75 mL butorphanol, and 19.75 mL saline. Each male IDOL mouse was intraperitoneally injected with 50 μL/g body weight of anesthetics 15 min before luminescence measurement. Immediately after measurement, each mouse was subcutaneously injected with 50 μL/g body weight of a solution consisting of 0.6 mL of the anti-anesthetic atipamezole and 9.4 mL saline. Mice were intraperitoneally injected with 0.1 μg/g body weight LPS and 1.0 μmol/g body weight KN3014 or DMSO. After 2 h, d-luciferin (Cayman Chemical, Ann Arbor, MI, USA) was injected intraperitoneally; 10 min later, luminescence signals were measured by AEQUORIA-2D/8600 (HAMAMATSU Photonics, Shizuoka, Japan). Whole-body images were saved as Tagged Image File Format (TIFF) files and opened with ImageJ software^33^. The threshold was adjusted for all samples, regions of interest (ROI) were determined, and histograms were analyzed.

### Isolation of splenocytes from IDOL mice

Spleens were harvested from IDOL mice, placed in 10 mL RPMI 1640 medium in a 100 mm plastic culture dish, and ground up using the rough side of an autoclaved slide glass. Whole cells were collected and passed through a 40 μm cell strainer (Greiner Bio-One GmbH, Kremsmünster, Austria) and collected in a 50 mL round tube. Cells were spun down, and the supernatant was removed. The cell pellet was resuspended (final concentration, 1 × 10^6^ cells/mL) for 4 h in RPMI 1640 medium containing 1.0 μg/mL LPS, and 1.0 or 10 μM KN3014 or KN8311.

### Cytokines secreted by cultured human peripheral mononuclear cells

Cytokine secretion assays were performed as described^25^. Briefly, PBMCs were cultured at a density of 1 × 10^5^/mL or 1 × 10^6^/mL in 1 mL RPMI1640 containing 10% heat-inactivated FBS per well of a 24-well flat-bottom plate (BD Biosciences, San Jose, CA, USA) in the presence or absence of lipopolysaccharides (LPS) from Escherichia coli O55:B5, cell-culture tested and purified by phenol extraction (Sigma-Aldrich, St. Louis, MO, USA) for 8 h at 37 °C in a humidified atmosphere with 5% CO_2_. Concentrations of IL-1β and TNF-α in the supernatants were measured by ELISA with specific antibodies (BD Biosciences)^25^.

### Evaluation of ASC-speck formation in peripheral blood mononuclear cells

ASC-speck formation was evaluated by immunofluorescence microscopy using anti-human ASC mAb. Briefly, separated human mononuclear cells were incubated with 0.1 ng/mL LPS plus KN3014, KN8311, MCC950, or vehicle (DMSO) for 8 h, and fixed with 70% ethanol at − 20 °C for 30 min. The fixed cells were incubated with anti-ASC mAb followed by goat-anti-mouse Alexa Fluor 488-conjugated secondary antibody (Jackson ImmunoResearch, 115-546-146).

### Evaluation of nuclear translocation of p65/NF-κB

Nuclear translocation of p65/NF-κB was evaluated by immunofluorescence microscopy using anti-human ASC mAb. Briefly, separated human mononuclear cells were incubated with 0.1 ng/mL LPS plus KN3014, KN8311, MCC950, or vehicle (DMSO) for 8 h, and fixed with 70% ethanol at − 20 °C for 30 min. The fixed cells were incubated with anti-p65/NF-κB mAb followed by goat-anti-rabbit Alexa Fluor 488-conjugated secondary antibody (Jackson ImmunoResearch, 111-546-144).

### Statistical analysis

All results are presented as the mean ± standard deviation (SD) of data from three independent experiments and compared by Mann–Whitney *U*-tests. A p-value < 0.05 was considered statistically significant.

### Ethics approval and guidelines for human experiments

Analysis of samples from the healthy volunteer and the patient were approved by the Human Research Ethical Committees of Ehime University (No. 1712006) and Shinshu University with the patient-supplying written informed consent (No. 476). All experiments were performed in accordance with the relevant guidelines and regulations for human samples.

All normal human PBMCs used in the study were separated from a healthy male volunteer. Human PBMCs from the healthy control and the patient with MWS were separated by Ficoll-gradient centrifugation, according to the manufacturer's instructions (GE Healthcare Biosciences AB, Piscataway, NJ, USA). The patient with MWS is a 21-year-old Japanese woman diagnosed with a CAPS/MWS at the age of 7 years; she harbors an arginine-to-tryptophan mutation at position 260 (R260W) of NLRP3^25^. She was being treated with injections of canakinumab (150 mg every 2 months); therefore, her PBMCs were separated just before injection to avoid the effects of medication.

### Ethics approval and guidelines for animal experiments

Mouse experiments were approved by the animal ethics committee of Ehime University and were performed in accordance with the relevant guidelines for care and use of animals.

## Supplementary information

Supplementary Information.

## Data Availability

All data generated or analyzed during this study are included in this published article.
