# Supplementary material for: KN3014, a piperidine-containing small compound, inhibits auto-secretion of IL-1β from PBMCs in a patient with Muckle–Wells syndrome
Source: Sci Rep. 2020 Aug 11;10:13562. doi: 10.1038/s41598-020-70513-0 (PMC7419506; doi:10.1038/s41598-020-70513-0)
Supplement: Supplementary file 1 — Supplementary Information. [file 41598_2020_70513_MOESM1_ESM.pdf]

KN3014, a piperidine-containing small compound, inhibits auto-secretion of IL-1β from PBMCs in a patient with Muckle-Wells syndrome

Naoe Kaneko[1], Mie Kurata[1], Toshihiro Yamamoto[1], Tomonari Shigemura[2], Kazunaga Agematsu[2,3], Takashi Yamazaki[2,4], Hiroyuki Takeda[5], Tatsuya Sawasaki[6], Tomohiro Koga [7,8], Atsushi Kawakami[7], Akihiro Yachie[9], Kiyoshi Migita[10], Koh-ichiro Yoshiura[11], Takeshi Urano[12] & Junya Masumoto[1]*

1) Department of Pathology, Ehime University Proteo-Science Center and Graduate School of Medicine, Shitsukawa 454, Toon, Ehime 791-0295, Japan.
2) Department of Pediatrics, Shinshu University Graduate School of Medicine, Asahi 3-1-1, Matsumoto, Nagano 390-8621, Japan.
3) Department of Infectious Immunology, Shinshu University Graduate School of Medicine, Asahi 3-1-1, Matsumoto, Nagano 390-8621, Japan.
4) Department of Pediatrics and Adolescent Medicine, Tokyo Medical University, Nishishinjuku 6-7-1, Shinjuku, Tokyo 160-0023, Japan.
5) Division of Proteo-Drug-Discovery Sciences, Ehime University Proteo-Science Center, Bunkyocho 3, Matsuyama, Ehime 790-8577, Japan.
6) Division of Cell-free Sciences, Ehime University Proteo-Science Center, Bunkyocho 3, Matsuyama, Ehime 790-8577, Japan.
7) Department of Immunology and Rheumatology, Division of Advanced Preventive Medical Sciences, Nagasaki University Graduate School of Biomedical Sciences, Nagasaki 852-8501, Japan.
8) Center for Bioinformatics and Molecular Medicine, Nagasaki University Graduate School of Biomedical Sciences, Nagasaki 852-8501, Japan.
9) Division of Medical Safety, Kanazawa University Hospital, Kanazawa, Ishikawa 920-8641, Japan.
10) Department of Rheumatology, Fukushima Medical University School of Medicine, Fukushima 960-1295, Japan.
11) Department of Human Genetics, Atomic Bomb Disease Institute, Nagasaki University, 1-12-4, Nagasaki 852-8523, Japan.
12) Department of Biochemistry, Shimane University School of Medicine, Izumo, Shimane 693-8501, Japan.
*Address correspondence and reprint requests to:
Junya Masumoto, MD, PhD
Department of Pathology

Ehime University Proteo-Science Center and Graduate School of Medicine
Shitsukawa 454, Toon, Ehime 791-0295, Japan
E-mail: masumoto@m.ehime-u.ac.jp
Phone: +81-89-960-5977
Fax: +81-89-960-5271

Keywords: cryopyrin-associated periodic syndrome, Muckle–Wells syndrome, NLRP3, inflammasome, interleukin-1β, small compound

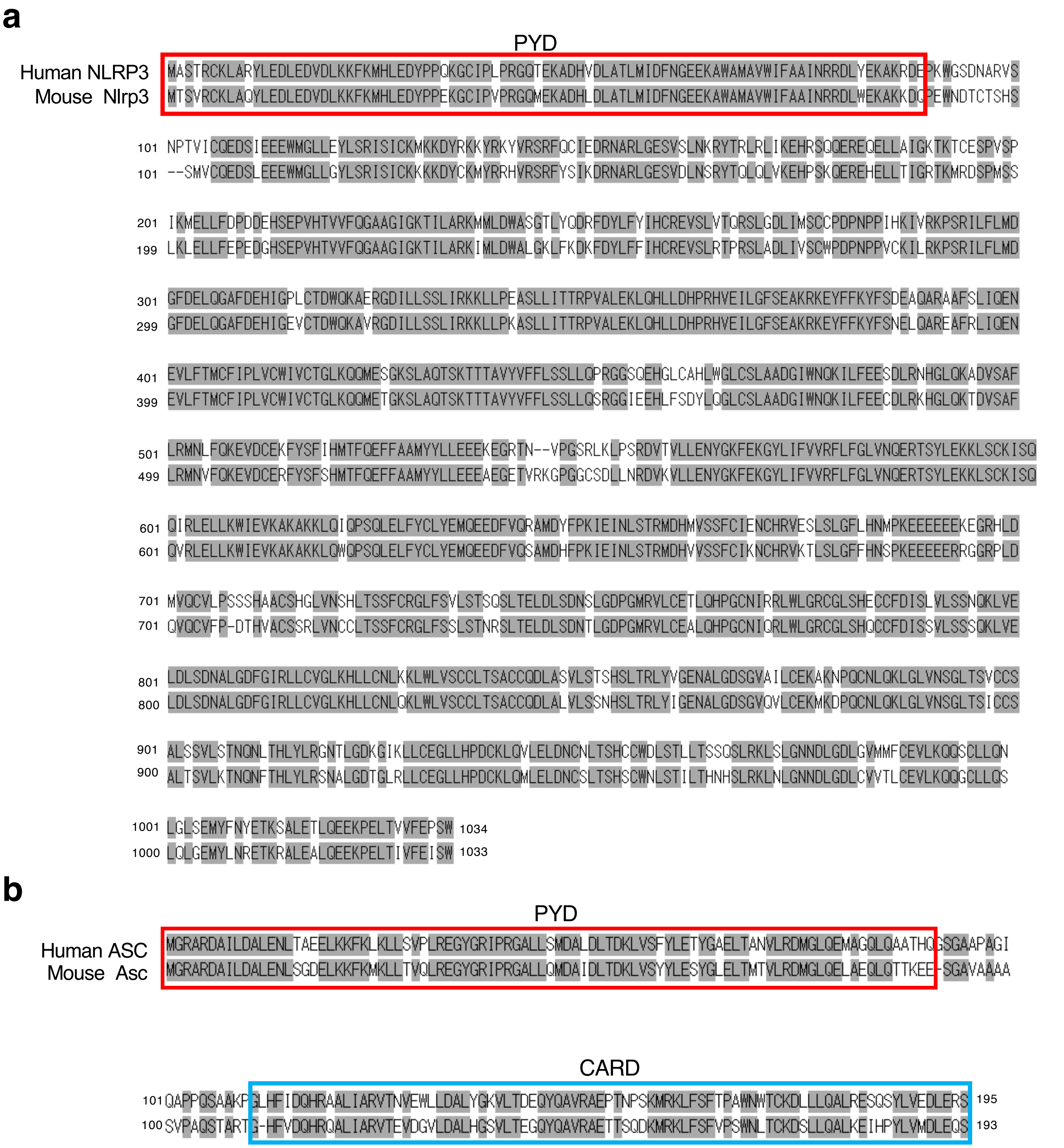

Supplementary Figure S1: Amino acid sequences of human and mouse NLRP3 (a) and ASC (b). The pyrin domains (PYD)s of NLRP3 and ASC are indicated by red boxes, and caspase recruit domain (CARD) of ASC is indicated by a blue box. Identical residues are shedded with gray.

1st screening

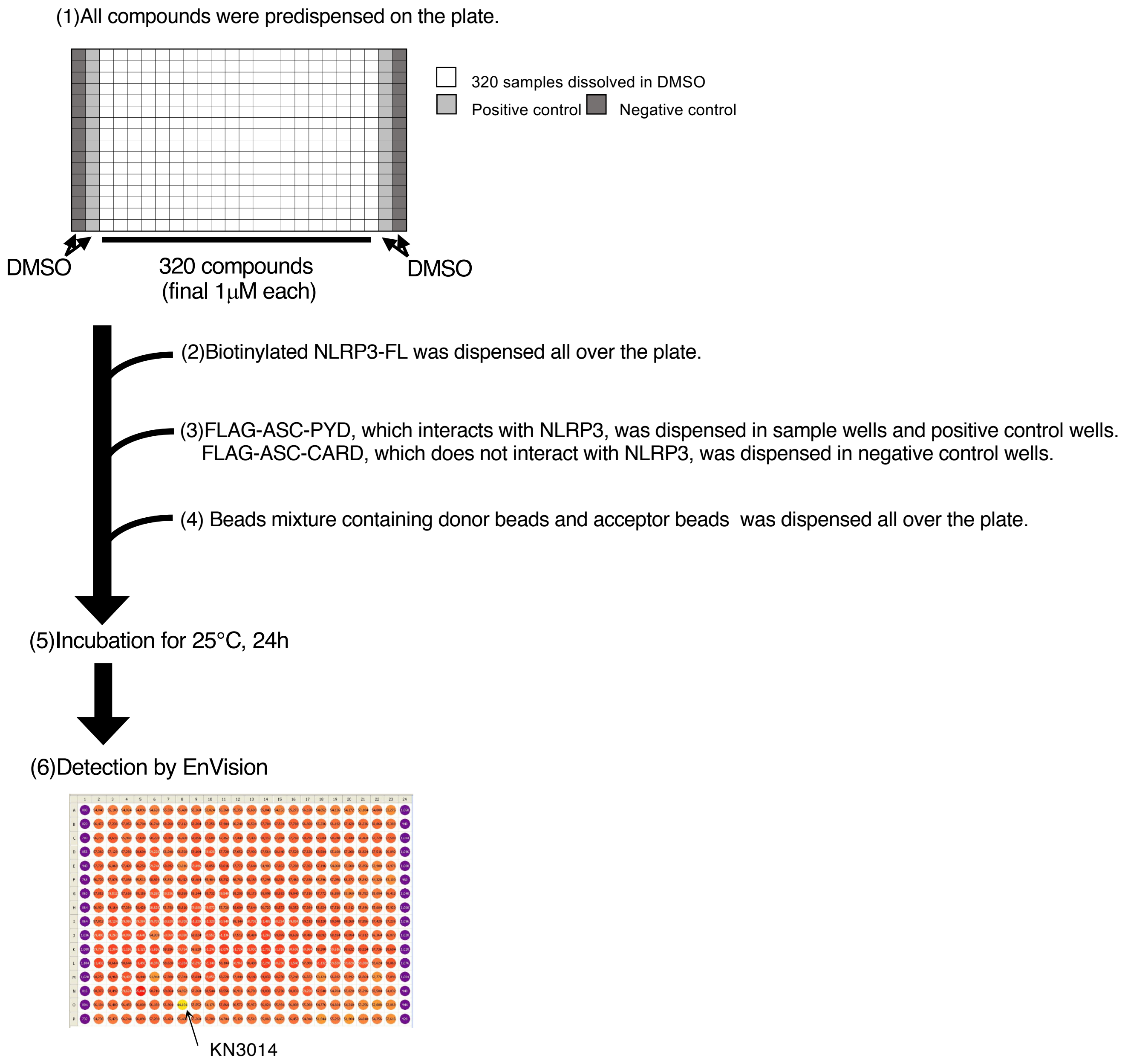

Supplementary Figure S2: Schematical representation of chemical screening. All compounds dissolved in DMSO were predispensed on an Optiplate-384 (250nL/well). (1)All compounds were predispensed on the plate. (2)Biotinylated NLRP3-FL was dispensed all over the plate. (3)FLAG-ASC-PYD, which interacts with NLRP3, was dispensed in sample wells and positive control wells. FLAG-ASC-CARD, which does not interact with NLRP3, was dispensed in negative control wells. (4) Beads mixture containing donor beads and acceptor beads were dispensed all over the plate. (5)Incubation for 25°C, 24h. (6)Detection by EnVision a multi-mode plate reader (PerkinElmer).

2nd screening

(1)Compounds were predispensed at the indicated concentration on the plate.

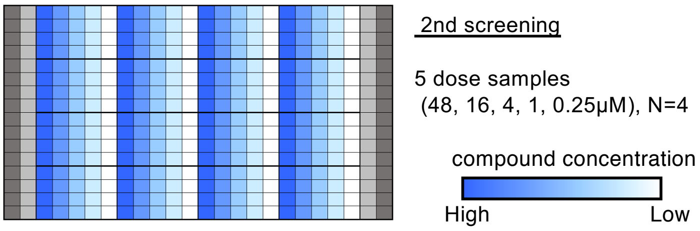

(2)Biotinylated NLRP3-FL was dispensed all over the plate.

(3) FLAG-ASC-PYD, which interacts with NLRP3, was dispensed in sample wells and positive control wells. FLAG-ASC-CARD, which does not interact with NLRP3, was dispensed in negative control wells.

(4) beads mixture containing donor beads and acceptor beads was dispensed all over the plate.

(5)Incubation for 25°C, 24h

(6)Detection by EnVision

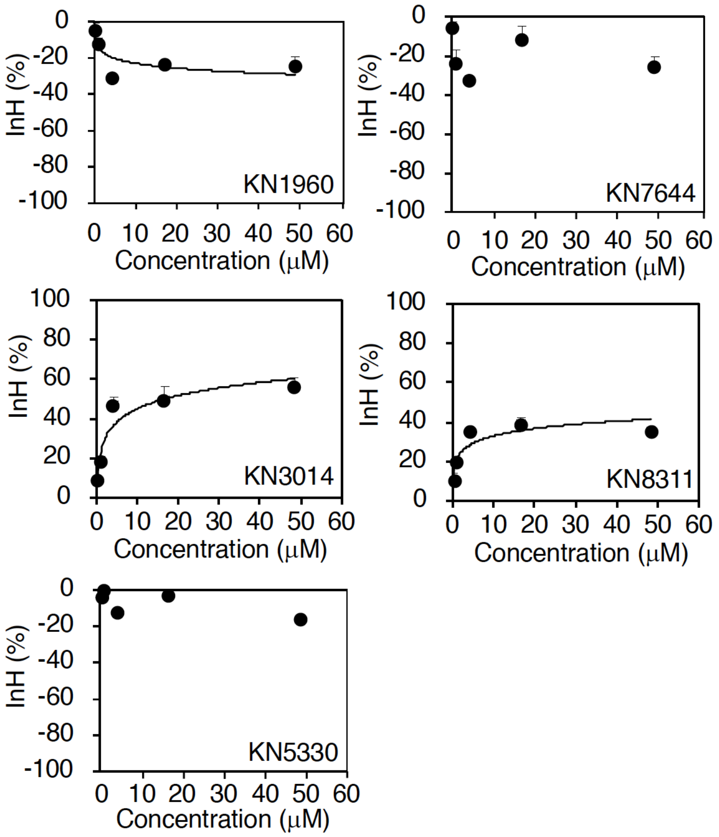

Supplementary Figure S3. 2nd screening was performed to confirm ability of candidate compounds. Chart of secondary screening using NLRP3 inflammasome in a cell-free system. The interaction between NLRP3-FL-Btn and FLAG-ASC-PYD was reduced or increased by KN1960, KN3014, KN5330, KN7644, or KN8311. The InH (%) of each compound is indicated on the y-axis. Compound concentrations are indicated on the x-axis. Results are given as means ± standard deviation of quadruplicate data. The data represent average values.

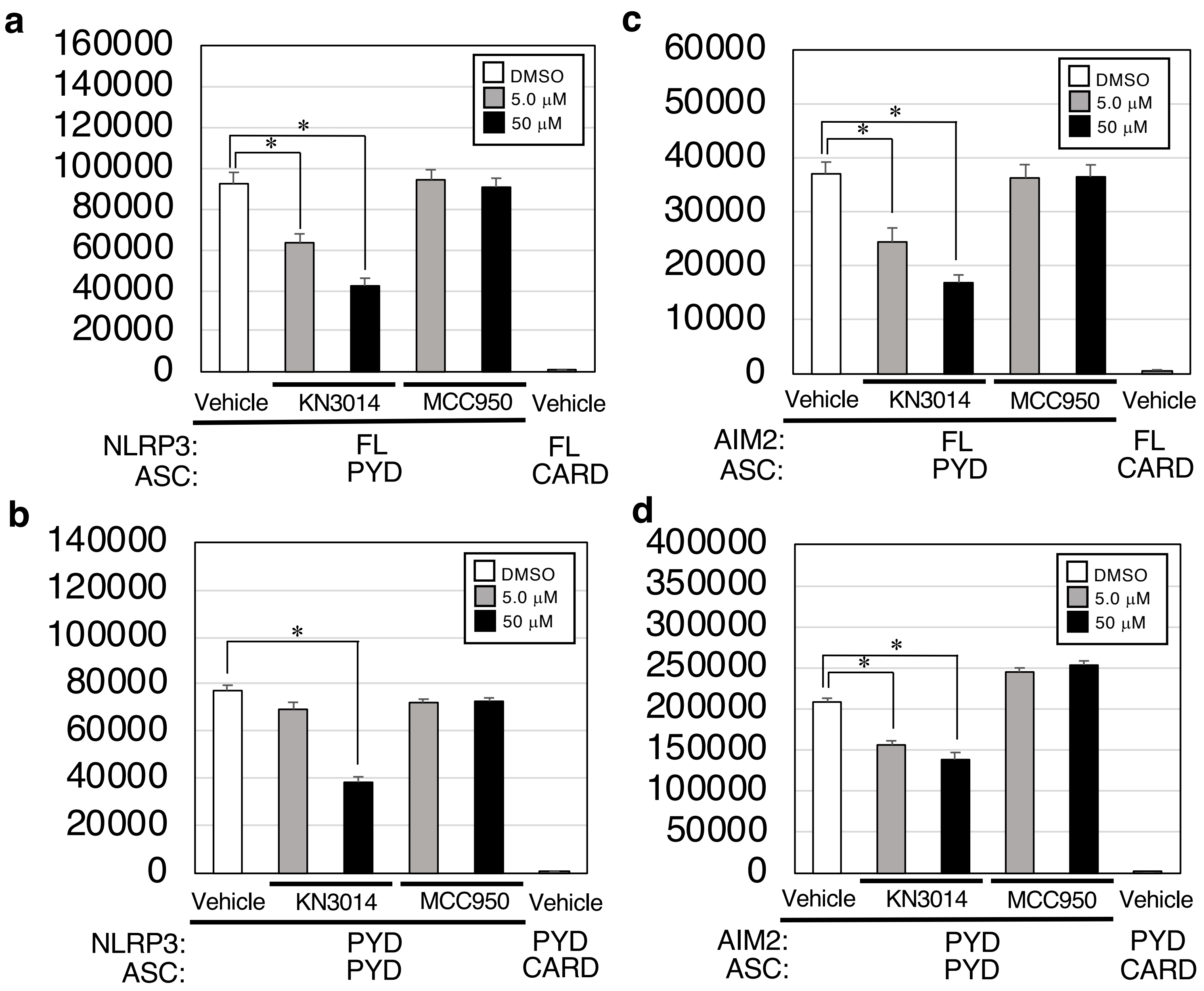

Supplementary Figure S4. 100 ng of full-length NLRP3 (NLRP3-FL) and pyrin domain of ASC (ASC-PYD) (a), or pyrin domain of NLRP3 (NLRP3-PYD) and ASC-PYD (b), or full-length AIM2 (AIM2-FL) and ASC-PYD (c), or pyrin domain of AIM2 (AIM2-PYD) and ASC-PYD (d) were incubated with 5 µg/mL anti-FLAG mAb M2, 16.67 µg/mL protein-A-conjugated Alpha acceptor beads and 16.67 µg/mL streptavidin-conjugated Alpha donor beads for 24 h with or without 5µM or 50µM, KN3014 or MCC950. Combinations of NLRP3-FL and caspase-recruitment domain of ASC (ASC-CARD) (a), NLRP3-PYD and ASC-CARD (b), AIM2-FL and ASC-CARD, and AIM2-PYD and ASC-CARD were negative controls. Results are given as means ± standard deviation of triplicate. *A p-value <0.05 was considered significant in a non-parametric statistical test the Mann-Whitney *U* test. These results are representative of two independent experiments.

a

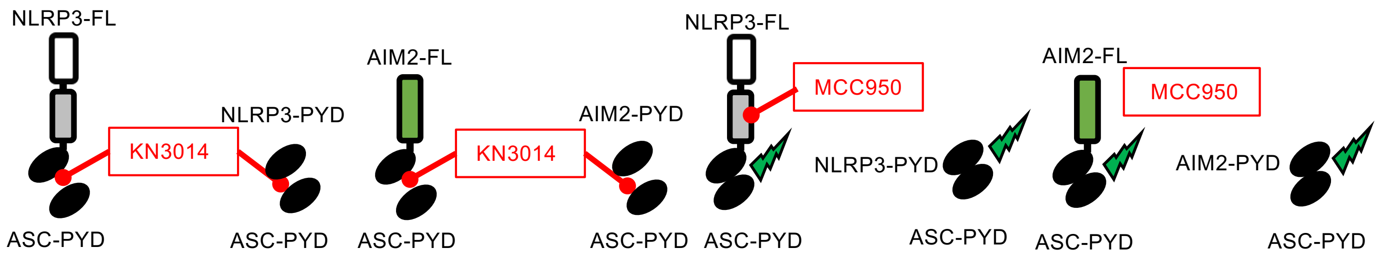

b

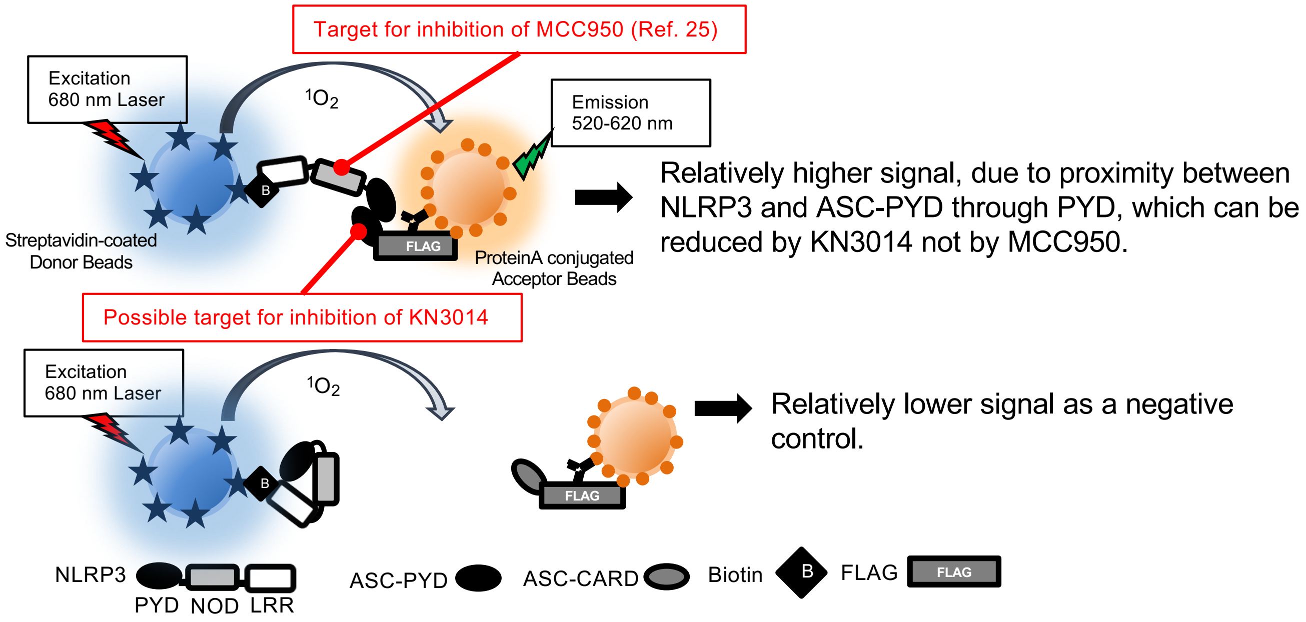

Supplementary Figure S5. Possible target of KN3014 against NLRP3 inflammasome. Since KN3014 reduced proximity signals between NLRP3-FL and ASC-PYD, NLRP3-PYD and ASC-PYD, AIM2-FL and ASC-PYD, and AIM2-PYD and ASC-PYD, that MCC950 did not reduced (a), target of KN3014 may be exist within ASC-PYD domain (b).

Supplementary Table S1. Precise ALPHA counts indicated in figure 2 and IC50.

Compound Name	Concentrtion (μM)	InH (%)	SD	IC50 (μM)
KN1960	48.33	-24.43	5.20	
KN1960	16.67	-23.87	2.24	
KN1960	4.17	-30.80	1.62	-
KN1960	1.00	-12.49	3.29	
KN1960	0.25	-5.07	4.22	
KN3014	48.33	56.42	4.45	
KN3014	16.67	49.60	6.91	
KN3014	4.17	47.02	3.97	14.65
KN3014	1.00	18.50	2.61	
KN3014	0.25	8.82	11.71	
KN5330	48.33	-16.49	6.12	
KN5330	16.67	-3.23	0.75	
KN5330	4.17	-12.95	3.24	-
KN5330	1.00	-0.04	4.59	
KN5330	0.25	-4.09	5.87	
KN7644	48.33	-25.60	5.06	
KN7644	16.67	-11.72	6.93	
KN7644	4.17	-32.60	2.45	-
KN7644	1.00	-23.53	6.45	
KN7644	0.25	-5.29	2.84	
KN8311	48.33	35.56	1.85	
KN8311	16.67	39.24	3.21	
KN8311	4.17	35.16	2.23	118.29
KN8311	1.00	19.61	1.65	
KN8311	0.025	10.26	3.92	

Footnote: InH, inhibition rate; SD, standard deviation; IC50, half maximal (50%) inhibitory concentration.
